# The OpenDeID corpus for patient de-identification

**DOI:** 10.1038/s41598-021-99554-9

**Published:** 2021-10-07

**Authors:** Jitendra Jonnagaddala, Aipeng Chen, Sean Batongbacal, Chandini Nekkantti

**Affiliations:** 1 School of Population Health, UNSW Sydney, Sydney, Australia; 2 School of Computer Science and Engineering, UNSW Sydney, Sydney, Australia; 3 CGD Health, Canberra, Australia

**Keywords:** Data mining, Machine learning

## Abstract

For research purposes, protected health information is often redacted from unstructured electronic health records to preserve patient privacy and confidentiality. The OpenDeID corpus is designed to assist development of automatic methods to redact sensitive information from unstructured electronic health records. We retrieved 4548 unstructured surgical pathology reports from four urban Australian hospitals. The corpus was developed by two annotators under three different experimental settings. The quality of the annotations was evaluated for each setting. Specifically, we employed serial annotations, parallel annotations, and pre-annotations. Our results suggest that the pre-annotations approach is not reliable in terms of quality when compared to the serial annotations but can drastically reduce annotation time. The OpenDeID corpus comprises 2,100 pathology reports from 1,833 cancer patients with an average of 737.49 tokens and 7.35 protected health information entities annotated per report. The overall inter annotator agreement and deviation scores are 0.9464 and 0.9726, respectively. Realistic surrogates are also generated to make the corpus suitable for distribution to other researchers.

## Introduction

Unstructured electronic health records (EHRs) such as discharge summaries, encounter notes, pathology reports and radiology reports are valuable sources of information for undertaking basic, clinical and translational research^1–3^. Often, researchers are required to share or access EHRs in a de-identified state to protect the privacy of patients. Traditionally, researchers manually redacted the Protected Health Information (PHI) in EHRs. It was reported that the average time required to manually de-identify a single clinical note (7.9 + /−6.1 PHI per note) was 87.3 + /−61 seconds^4^. It is practically not possible to de-identify large numbers of EHRs manually. Identifying PHI in unstructured EHRs is also critical for electronic phenotyping and record linkage^5,6^. Furthermore, manual de-identification is not possible to integrate into routine clinical workflows. For example, in biobanking systems, the pathology reports are automatically obtained once the patient agrees for biospecimen collection. These reports are then delivered directly to the biobanking management system. However, the reports need to be de-identified in order to be shared with the researchers looking for specific biospecimens. Similarly, there are many other clinical workflows, where de-identification of these unstructured EHRs is vital. Over the years, several studies have been conducted on de-identification of EHRs using manual, rule based and machine learning methods. Automated de-identification methods can be employed to replace manual processes^1,7–11^. These methods, especially deep learning-based methods, often require a large corpus for accurate de-identification.

A de-identification corpus is a large set of unstructured texts with PHI entities that has been manually annotated. The corpus annotation process requires careful design and execution of a systematic approach. Failing to appropriately annotate the corpus may lead to inaccurate predictions by automated de-identification systems compromising patient privacy. The process of annotating a corpus for de-identification purposes is similar to the process used in named entity recognition (NER) tasks. Individual PHI entities are identified and categorised into pre-defined categories^12^. When constructing a corpus for de-identification, the goal is to locate and classify any PHI that exists in clinical texts and preserve as much data as possible. De-identification differs from other NER tasks such as disease and drug recognition^13^. De-identification has little dependency on clinical context but strong dependency on organizational context. Several corpora of unstructured EHRs exist for clinical NER tasks. The Clinical E-Science Framework project shared a corpus containing 565,000 clinical records from 20,000 patients including clinical narratives, histopathology reports and imaging reports^14^. The Text Retrieval Conference also shared a corpus with 17,000 records to offer health records based on the semantic content of free-text fields^15^. However, given the complex nature of automatic de-identification tasks, very few corpora are available for the development of automatic de-identification systems^16,17^.

The 2014 i2b2/UTHealth de-identification corpus contained a total of 1,304 longitudinal clinical narratives of 296 patients from USA. In this corpus 28,872 PHI were annotated and classified into 6 PHI categories and 25 subcategories^17,18^.Another corpus is 2016 CEGS N-GRID de-identification corpus of 1000 psychiatric notes from USA^19^. Deleger et al. (2014) also created a corpus of 3,503 medical records with 30,815 PHI entities annotated. The corpus contains 22 different types of clinical notes such as operative, pharmacy, progress, and discharge notes. However, the corpus did not include any pathology reports^20^. Uzuner et al. (2007) also shared a corpus consisting of 889 hospital discharge summaries, containing a total of 19,498 PHI in 8 main categories and 25 subcategories^13^. In a recent study ^21^, the Medical Information Mart for Intensive Care III (MIMIC-III) database ^22^ is used to semi-automatically construct a corpus using the PhysioNet DeID tool^9^. Annotators have manually reviewed and improved the PhysioNet DeID tool annotations.

Traditional rule-based de-identification methods rely heavily on dictionary lists and gazetteers. PHI that are ambiguous and not covered in dictionary lists or gazetteers decrease the performance of rule-based de-identification methods. Furthermore, the presence of many categories in the PHI also increases the difficulty in de-identification using machine learning based methods. Similar corpora are constructed from other countries to fit local use^23,24^. All these corpora employ various annotation approaches. Uzuner et al. (2007) employed serial annotation by two annotators^11,13^. Deleger et al.^20^used parallel annotations and Stubbs et al. (2015) employed serial annotations, parallel annotations and pre-annotations^17^. Similar types of annotation settings are used in the construction of PGx corpus of PubMed abstracts^25^ and CHIA corpus of clinical trial eligibility criteria^26^. In an another similar study, Spanish clinical trial studies announcements are pre-annotated using a hybrid approach^27^. Though there are many similar corpora, in a few studies the ability of automated pre-annotations to improve the performance of manual annotation is evaluated^27–30^.

As per our knowledge at the time of this publication, there is no corpus available from Australia for the purpose of automatic de-identification. Most of the existing corpora available in Australia are constructed using pathology reports for clinical NER tasks and are not related to de-identification^31^. Though there are few de-identification corpora available outside Australia, their performance is significantly reduced when applied on Australian EHRs. For example, Australian hospital names, cities, and other location specific entities are not likely to exist in corpora constructed using EHRs from other countries. Patterns and sequences such as phone numbers, postal codes, and ID including social security numbers, medical record numbers might also vary from other countries. There is very limited evidence around this research gap with only a handful of studies. In Zuccon et al. (2013) an automatic de-identification system was developed using machine learning techniques using 669 documents from the 2006 i2b2 de-id corpus and tested on 228 Australian pathology reports^32^. The portability of the model to Australian EHRs was noted to be poor (F-measure of 0.286). It is of great importance to construct a robust de-identification corpus that is suitable for Australian context. Additionally, there is limited evidence regarding the impact of annotation approach on time and quality. The quality of annotations in serial and parallel orders were compared in a previous study but the time aspect of the annotation process was not investigated^28^. In this study we aim to construct a large corpus of pathology reports for automatic patient de-identification. Furthermore, we aim to investigate the cost and quality of corpus annotations under three different settings using different annotation approaches.

## Results

The final gold standard OpenDeID corpus consists of 2,100 unique pathology reports of 1,833 unique cancer patients from four urban Australian hospitals. The corpus consists of 38,414 PHI entities and 1,548,741 tokens (Table 1). The average number of tokens and PHI entities per report were 716.88 and 18.29, respectively. The distribution of PHI entities across different category types is even in all three settings. Most of the annotated PHI entities belong to NAME category, followed by LOCATION, ID and DATE. AGE and CONTACT contributed a small fraction to the corpus, while PROFESSION and OTHER categories did not appear in our corpus. A detailed distribution over PHI subcategories is presented in Supplementary Table 3.

**Table 1:** Summary of the OpenDeID corpus where n = total number of pathology reports.

Category	All settings (n = 2100)	Setting 1 (n = 700)	Setting 2 (n = 700)	Setting 3 (n = 700)
NAME	11,789	3929	3903	3957
AGE	141	40	42	59
CONTACT	7	1	5	1
LOCATION	9861	3359	3151	3351
DATE	7665	2566	2501	2598
ID	8951	2913	3042	2996
PROFESSION	0	0	0	0
OTHER	0	0	0	0
Total number of PHI entities	38,414	12,808	12,644	12,962
Average number of PHI entities per report	18.29	18.29	18.06	18.51
Standard deviation of PHI entities	7.35	6.85	7.67	7.50
Total number of tokens	1,548,741	510,357	508,988	529,396
Average number of tokens per report	737.49	729.08	727.12	756.28
Standard deviation of tokens	362.33	345.18	374.74	366.22

The total time spent by annotators in Setting 2 is 55.2 h (Table 2), the highest among the three settings. Annotators in Setting 1 spent 17.8 h less than Setting 2. In Setting 3, the total time spent by annotators is 27.75 h, the lowest of all settings, almost half the time spent in Setting 2. The average time spent per report in all settings is 10.31 min. However, the average time spent for each report in the training phase is 27.1 min.

**Table 2:** Time spent under each annotation setting.

Setting	Annotator	No. of reports	Time spent by annotators independently (hours)	Time spent by annotators collaboratively (hours)	Total time (hours)
1	1	700	24.65	8.25	37.4
	2		4.5		
2	1	700	25.9	9.75	55.2
	2		19.55		
3	1	700	10.8	9.75	27.75
	2	700	7.2		

The Inter Annotator Agreement (IAA) and Deviation Score (DS) under all three settings are presented in Tables 3 and 4, respectively. The overall IAA reached 0.9464 amongst all three settings. The IAA for Setting 1 and Setting 2 are 0.9559 and 0.9337, respectively. However, Setting 3 achieved the lowest IAA of 0.8721 and 0.8999. Under Setting 3, Recall was significantly lower than Precision and F-measure. In Setting 3, NAME and ID categories had low IAA and DS. Supplementary Table 4 and Table 5 present detailed IAA and DS for each PHI category under each setting. Annotation quality varied across the PHI categories. The IAA and DS are > 0.95 in most categories under each setting except in LOCATION and AGE categories. Discrepancies in annotations across these two categories are noted to be common in all 3 settings, with IAA < 0.9. The DS of Annotator 1 is also relatively low when compared to Annotator 2 for these two categories in all settings. The DS of Annotator 1 for LOCATION was the lowest across all settings. The DS of Annotator 2 across all settings and categories remained consistent except for CONTACT category, which had very low count on entities in the corpus. In general, Annotator 1 had lower IAA and DS when compared to Annotator 2.

**Table 3:** IAA across individual settings.

	All settings (n = 2100)	Setting1 (n = 700)	Setting2 (n = 700)	Setting3 PhysioNet DeID vs Annotator1 (n = 700)	Setting3 PhysioNet DeID vs Annotator2 (n = 700)
Precision	0.9482	0.9565	0.9329	0.9263	0.9618
Recall	0.9445	0.9552	0.9346	0.824	0.8455
IAA	0.9464	0.9559	0.9337	0.8721	0.8999

**Table 4:** DS across individual settings.

	All settings (n = 2100)		Setting1 (n = 700)		Setting2 (n = 700)		Setting3 (n = 700)	
	Annotator1	Annotator2	Annotator1	Annotator2	Annotator 1	Annotator 2	Annotator 1	Annotator 2
Precision	0.954	0.997	0.9564	0.9998	0.9508	0.991	0.9549	0.9934
Recall	0.9466	0.9931	0.9552	1	0.9411	0.979	0.9436	0.9934
DS	0.9503	0.995	0.9558	0.9999	0.9459	0.985	0.9492	0.9934
Average DS	0.9726		0.9779		0.9655		0.9713	

Supplementary Table 5 presents the p-values of the significance tests for time and quality metrics across all three settings. The pairwise comparison of time metric across all settings is found to be statistically significant (p-value < 0.0001) except between Setting 1 and Setting 2 (p-value < 0.0667). In other words, using PhysioNet DeID for pre-annotations has significantly reduced the total time taken in Setting 3. The differences in overall IAA among all three settings are statistically significant, mainly in NAME, LOCATION, DATE, and ID categories. However, there is no statistically significant differences in IAA for AGE across all the settings. Like the time metric, IAA has significantly decreased in Setting 3 PhysioNet DeID when compared to Setting 1 (p-value < 0.0001) and Setting 2 (p-value < 0.0001) across all PHI categories except for LOCATION. A statistically significant difference between DS in all settings is observed (< 0.0001). DS between Setting 1 and Setting 2, and between Setting 1 and Setting 3 is significant but between Setting 2 and Setting 3 is not (p-value < 0.2981).

Our results suggest that although time can be decreased with pre-annotations using automated rule-based de-identification systems, the quality of the corpus could decline when compared to serial annotations. Our results are congruent with previous findings, that automatically pre-annotating corpus can significantly save time while there is no significant difference of annotation quality between parallel and pre-annotations^30,33^. Comparison between Setting 1 and Setting 2 suggests that the former has better quality, contrary to what is observed in a previous study^28^. For construction of de-identification corpus, we recommend using Setting 1. Setting 1 is an optimal choice in terms of time and quality^34^.

## Discussion

We constructed a large gold standard annotated corpus of 2,100 unstructured pathology reports retrieved for automatic patient de-identification. We evaluated the time and quality of annotations by two annotators under three different settings. Time and quality are intertwined aspects of great importance in corpus construction. The time spent is an important comparison factor that governs cost economics, which in turn can justify the compromise on quality. Thus, we need to find a setting that provides good quality annotations in a reasonable time frame which would equate to a reasonable cost.

We found Setting 1 and Setting 3 more effective than Setting 2. This is due to the large number of conflicting annotations that needed to be resolved in Setting 2. This shows great potential of pre-annotations in improving the efficiency of the corpus annotation process, in both time and quality. In our case, pre-annotations improved the overall speed of annotating our corpus but there was not much difference in quality. However, depending on the automatic tool used, pre-annotations might have a negative impact on quality. Setting 3 is not generalisable and performance depends on the system and corpus used to generate pre-annotations. It is also important to factor in the additional time required in Setting 3 for tuning the performance of automated system. For example, in our study we improved PhysioNet DeID tool to support Australian based PHI entities. This is reflected in our significance tests, which showed no statistically significant difference for the LOCATION category. In our study we assumed the time required to configure, improve, and use PhysioNet DeID tool as minimal.

Pre-annotations using machine learning based approaches seemed to increase quality but did not decrease overall time required in a French de-identification corpus^24^. The quality and time is found to be insignificant in a different English based de-identification corpus^30^ using a hybrid system^35^. Time saved via pre-annotations with rule-based dictionaries in a NER task which is not related to de-identification is found to be significant^29^. Similar results are observed in our study. The total time spent is significantly (p-value < 0.0001) less in Setting 3 (27.75 h), which used rule-based PhysioNet DeID tool, when compared to Setting 1 (37.4 h) and Setting 2 (55.2 h). It is worth noting that Annotator 2 annotated files under Setting 2 and reviewed the results under Settings 1 and 3. Thus, the time spent is significantly less for Annotator 2 under Setting 1 and Setting 3 compared to Annotator 1. Annotator 2 was more experienced in annotation tasks, as a result spent less time in Setting 2 when both annotators were involved in the annotation tasks. It is important to recognise the differences in experimental settings and context of Grouin et al. (2014), Lingren et al. (2014), South et al. (2014) and our study^24,29,30^. These experimental design and contextual differences do not permit direct comparisons. For example, in Grouin et al. (2014) sequential pre-annotations are spread over various iteration cycles^24,29^. This allows machine learning-based pre-annotations to improve after every iteration cycle. Similarly, in Lingren et al. (2014) the NER task is disease and symptom recognition in clinical trial announcements, which do not include PHI^29^. Therefore, results presented in this study need to be interpreted judicially. Active learning is another approach that can be employed for pre-annotations. Boström and Dalianis (2012), employed active Learning to de-identify 100 Swedish EHRs^36^. In a more recent study, using the 2006 i2b2 dataset, Li et al. (2019) established that small number of annotated documents are required to reduce the annotation workload using active learning^37^.

The DS of Annotator 1 remained consistent and did not vary across all the settings. However, the performance of Annotator 2 varied under Setting 1 and Setting 2. This can be attributed to the fact that Annotator 2 in Setting 1 reviewed Annotator 1’s annotations whereas in Setting 2, Annotator 2 independently performed the annotation. In the categories of NAME, LOCATION, DATE and ID, Annotator 2’s DS showed significant improvement under Setting 1 compared to Setting 2. The DS for both annotators in Setting 3 for NAME category was lowest when compared to remaining categories. This suggests that PhysioNet DeID tool had trouble identifying names related to Australian healthcare context. Annotators had a higher agreement in the NAME category under Setting 1 and Setting 2 with an IAA of 0.9971 and 0.9785, respectively. After review, the quality of NAME annotations in Setting 3 improved to that of Setting 1 and Setting 2. ID category entities were also difficult for the PhysioNet DeID to recognize. We believe that it was because the ID entities do not have a fixed pattern, making it difficult for rule-based systems such as PhysioNet DeID to recognise. It can be seen from Supplementary Table 5 that Annotator 1 missed more ID than Annotator 2. Though annotators went through an iterative training phase, in a few situations they deviated from the guidelines. For example, for the LOCATION category only one unique occurrence was supposed to be annotated. Annotator 1 marked all observed LOCATION entities in each document, which led to a higher disagreement with Annotator 2 and the final gold set in all three settings.

This corpus was constructed specifically for an automated de-identification task. However, in the future we intend to annotate the OpenDeID corpus with disease, drug, and procedure entities. As such the OpenDeID corpus for other purposes apart from automated de-identification, disease, drug and procedure entity recognition and normalisation is not recommended. Surrogate generation of few entities such as age and dates reduce the reusability of the corpus for secondary clinical, molecular, or epidemiological investigations. Additionally, this corpus contains cancer biobanking related pathology reports, and as such, performance of automated de-identification systems trained on other types of clinical documents such as discharge summaries and clinical narratives may vary. However, for the patient de-identification task we hypothesise the performance difference will not be significant. The performance of multiple automated de-identification systems on the OpenDeID corpus is yet to be evaluated.

## Methods

Reports (n = 4,548) in the form of HL7 messages and 156 reports in the form of PDF were retrieved from four urban Australian hospitals. Reports were excluded if there was a low token count (n = 2,162), and if the reports were not pathology reports or if the reports had inconclusive results (n = 292). The final set to be annotated comprised of 2,100 reports from 1833 unique cancer patients. The complete cohort selection process of the OpenDeID corpus is explained elsewhere^38^. Individual patient informed consent was obtained through the Health Sciences Alliance (HSA) biobank. The HSA biobank is an institutional biobank based at the Lowy Cancer Research Centre at the University of New South Wales, Sydney, Australia^39,40^. The patient consent covers data linkage with other data sources for future research and ongoing storage of biospecimens. We have retrieved surgical pathology reports of cancer patients to construct the OpenDeID corpus, presented in this study. Ethics to develop this corpus was approved by the UNSW Sydney Human Research Ethics committee (approval no. HC17749). This research was undertaken in accordance with the approved ethics application, relevant guidelines, and regulations.

### Corpus construction

**Figure 1:**
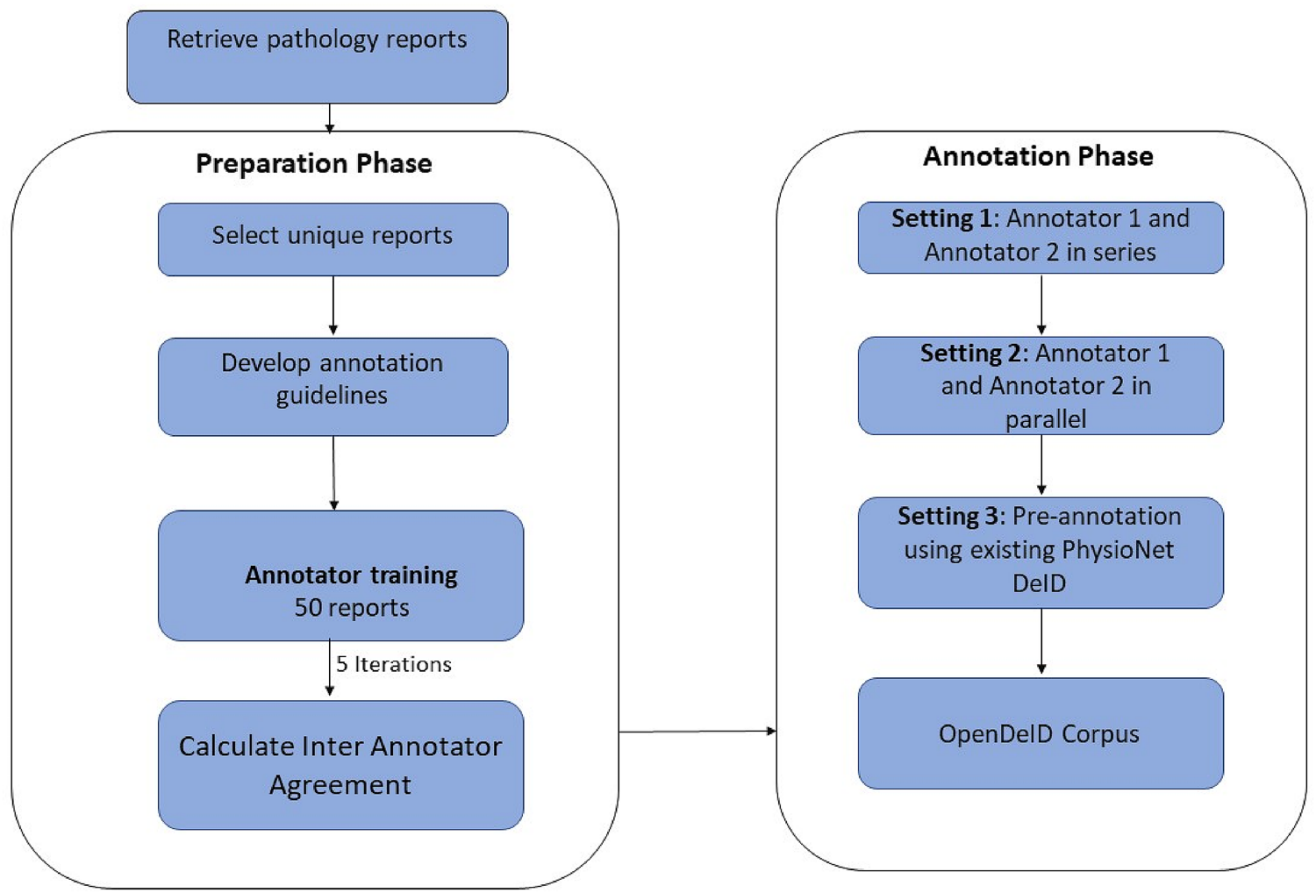
Overall OpenDeID corpus construction process.

The overall corpus construction process was carried out in two phases (Fig. 1). The first phase was the preparation phase, followed by the annotation phase. In the preparation phase, we retrieved and examined the pathology reports to understand syntactic and semantic content. This was followed by setting the process of annotating the extracted reports. We used the MAE (Multi-document Annotation Environment) v2.1.3 tool, which is a general-purpose annotation tool^41^. The output files were in XML format as per the document type definition (DTD) designed in phase 2. Each XML file has 2 main elements called TEXT and TAGS. The TEXT element contains the original content of the report. The TAGS element contains annotations that were marked within the original text. Each annotation is a child of the TAGS element. Each annotation element itself was made up of several attributes such as offsets, categories, subcategories, and comments.

### Preparation phase

We have adapted existing PHI annotation guidelines and improved them to suit our requirements^17^. In addition to the HIPAA PHI entities, we have added additional PHI entities which potentially include identifiable information. Additional PHI entities included indirect information such as names of hospitals, doctors, nurses; dates; location information; and patients’ professions. Patients’ age, irrespective of age group, is considered as a PHI. These PHI entities have been grouped into 8 unique categories and 27 unique subcategories, such that we can use this granular information for de-identification. Supplementary Table 1 shows the categories, subcategories, and examples of PHI entities. We developed a guideline that contained annotation instructions along with several examples. The last stage of this phase was training the annotators. The training was carried out in five iterations. In each iteration, a set of 50 reports (that are not part of the final 2,100 reports) were annotated by the annotators. Reports with token counts within the range of 700–1200 were selected. Feedback from each iteration was provided to the annotators to help them better understand the annotation guidelines and improve the quality of annotations. IAA was calculated between both annotators to assess the quality. Once IAA exceeded 0.8, the annotators were deemed eligible for the final annotation.

### Annotation phase

**Figure 2:**
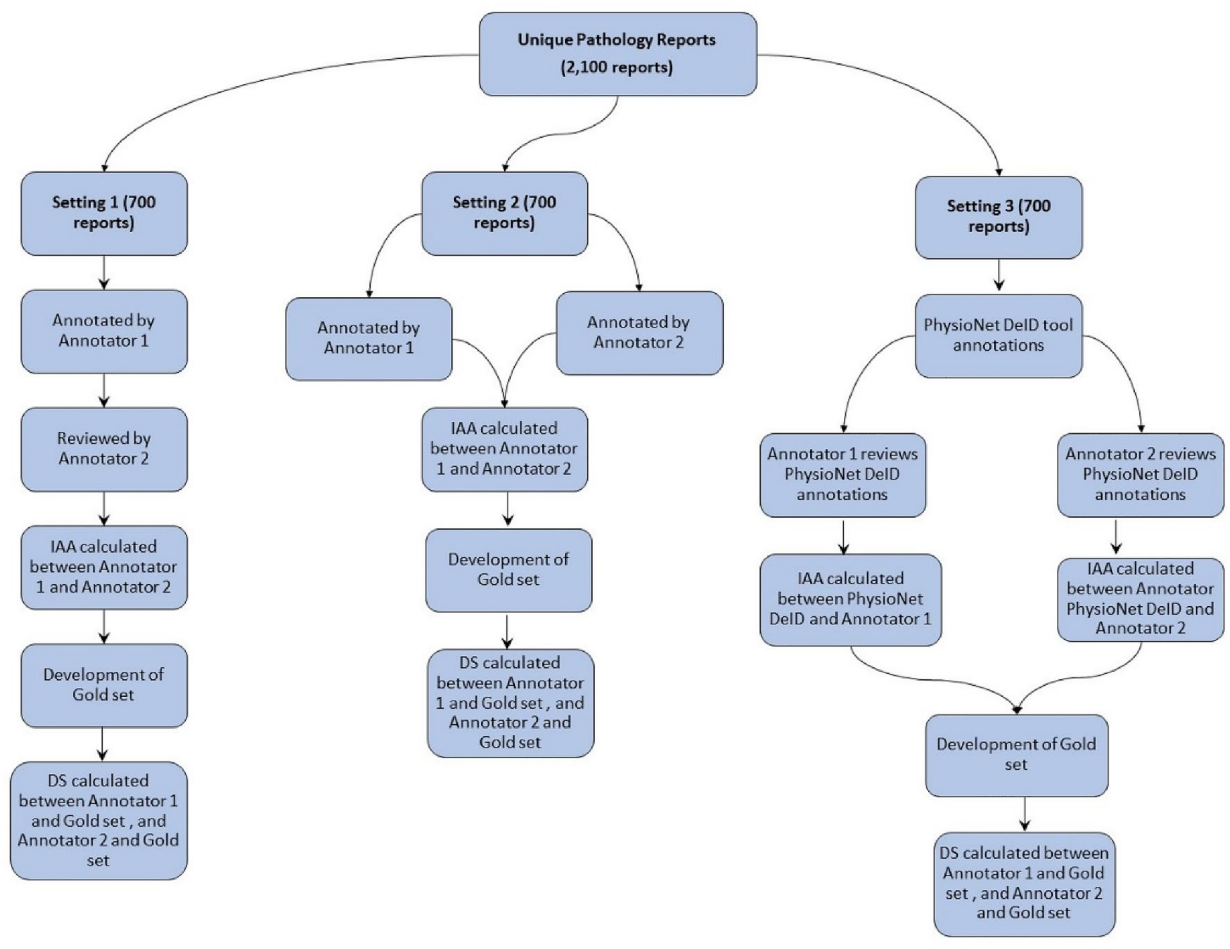
Annotation process under three different settings.

The annotation phase was carried out under three different settings (Fig. 2) in batches. Each batch consisted of 50 reports. 2,100 pathology reports were randomly divided into three equal subsets. In Setting 1, Annotator 1 annotated the first 700 reports. Then, Annotator 2 reviewed Annotator 1’s annotations and made necessary corrections. This was followed by the calculation of IAA. In the next step, the gold set was developed. DS was then calculated between the final gold set and each annotator’s annotation. In Setting 2, both the annotators independently annotated the second subset of 700 reports. IAA was then calculated between the annotators. Then, like the previous setting gold set was prepared and DS calculated. In Setting 3, PhysioNet DeID tool^9^ was used for the remaining 700 reports. PhysioNet DeID tool is a rule-based system that can detect HIPAA PHI entities using pre-built dictionaries and lists of PHI entities. Though the tool has been primarily developed using nursing notes from USA, it has been applied to discharge summaries and other type of unstructured EHRs^42^. We have improved the tool by enriching the dictionaries and gazetteers to include Australian PHI entities. The annotations were then reviewed and refined by Annotator 1 and Annotator 2 independently, followed by IAA calculation, gold set development and DS calculation.

In all settings, the gold set was developed by combining annotations from Annotator 1 and Annotator 2. In situations of conflicting annotations, Annotator 1 and Annotator 2 discussed each conflict and reached a consensus. Both annotators have post graduate qualifications in medicine and are authorised to access the reports. Annotations from Annotator 1 and Annotator 2 were verified by all the co-authors during the training process. Additionally, 5% of annotations were independently verified in each setting by all the co-authors. As part of the gold set creation realistic surrogates were also generated. Generation of realistic surrogates is required to protect the privacy of patients and to maintain integral characteristics of the corpus. We have described our surrogate generation process in-detail elsewhere^43^. After replacing the PHI with realistic surrogates, the corpus was further verified manually by both annotators for any possible PHI. In situations where automatic surrogate generation failed, surrogates were generated manually.

### Corpus assessment

A comparison of the annotations was performed on two dimensions: time and quality. The time spent on annotation for each batch (50 reports) was tracked by the annotators. These logs were summed up to obtain the total time spent annotating all files under each setting. To compare the quality, IAA and DS were calculated (Supplementary Table 6A, 6B). IAA is a measure of how well the two annotators can make the same annotation decision. IAA provides an insight on the similarity of the annotations between two annotators. The score is calculated for 2 annotators as Annotator 1 vs Annotator2. The higher the IAA scores are, the better is the agreement and compliance with the annotation guidelines. This in turn results in high quality annotations^44^. DS is defined as the difference between the final gold set and each annotation. Thus, within each setting there were 2 scores. DS for Annotator 1 was defined as gold vs Annotator 1, and DS for Annotator 2 was defined as gold vs Annotator2. IAA can be considered as interim quality of annotations and DS as the overall quality of the annotations. In NER tasks such as de-identification, F-measure is recommended to assess the quality of corpus^45^. We evaluated IAA and DS using F-measure as a surrogate for Kappa or weighted kappa. Specifically, we used micro-averaged strict type of F-measure to report IAA and DS. Corresponding Precision and Recall were also calculated at category and subcategory levels^17,19^. Overall PHI and category-wise PHI significance tests were performed to compare time and quality metrics across the three different annotation settings. We adopted one-way analysis of variance (ANOVA) with Bonferroni correction to determine if the variance between each setting was statistically significant. A p-value < 0.05 was considered statistically significant difference between settings^46^.

## Conclusion

The OpenDeID corpus is the largest Australian corpus of unstructured EHRs available for development of automated de-identification systems. The corpus comprises 2,100 pathology reports from 1833 patients from four urban hospitals with 38,414 PHI entities. Our experiences suggest that annotating with two annotators is a balanced approach in terms of cost and quality. Among the three different annotation settings, we found that the most efficient setting in terms of time and quality is, the setting where two annotators annotated the corpus in serial. This setting is time saving and with non-significant loss of quality when compared to the other two settings. Semi-automated pre-annotations are effective in reducing annotation time but are not generalisable. They are highly dependent on the performance of the automated system used, which sometimes may deteriorate the quality of pre-annotations causing increase in time required by the annotators to review and resolve conflicts.

## Supplementary Information

Supplementary Information.

## Data Availability

The instructions to access the OpenDeID corpus are available at https://github.com/TCRNBioinformatics/OpenDeID-Corpus. Additionally, the annotation guidelines; code used to, select the cohort, generate the DS and IAA metrics, and evaluate performance can be made available up on request.
