# Supplementary material for: The OpenDeID corpus for patient de-identification
Source: Sci Rep. 2021 Oct 7;11:19973. doi: 10.1038/s41598-021-99554-9 (PMC8497517; doi:10.1038/s41598-021-99554-9)
Supplement: Supplementary file 1 — Supplementary Information. [file 41598_2021_99554_MOESM1_ESM.docx]

**SUPPLEMENTARY INFORMATION**

| **PHI category** | **Subcategory** | **Example** |
| --- | --- | --- |
| NAME | PATIENT, DOCTOR, USERNAME | John Doe, Dr. Max, Mr. Smith |
| PROFESSION | NONE | Lawyer, teacher |
| LOCATION | ROOM, DEPARTMENT, HOSPITAL, ORGANIZATION, STREET,  CITY, STATE, COUNTRY, ZIP, OTHER | PERI-OPERATIVE UNIT-POW, MACQUARIE WARD – RHW,  12 ABC Street |
| AGE | NONE | 23, 98 |
| DATE | NONE | 24/12/1987, September 26th |
| CONTACT | PHONE, FAX, EMAIL,   URL, IPADDRESS | [abc@gmail.com](mailto:abc@gmail.com),  194.223.1.1 |
| ID | SOCIAL SECURITY NUMBER,  MEDICAL RECORD NUMBER,  HEALTH PLAN NUMBER,  ACCOUNT NUMBER,  LICENSE NUMBER,  VEHICLE ID,  DEVICE ID,  BIOMETRIC ID,  ID NUMBER | MRN:  9174338  ID NUMBER: 12R1500257 |
| OTHER | NONE | Fingerprint, Company Logo |

Supplementary Table 1. Detailed PHI categories and subcategories with examples

| **PHI category: Subcategory** | **Entity Counts** |
| --- | --- |
| NAME: PATIENT | 2118 |
| NAME: DOCTOR | 9671 |
| NAME: USERNAME | 0 |
| PROFESSION | 0 |
| LOCATION: ROOM | 1 |
| LOCATION: DEPA RTMENT | 1840 |
| LOCATION: HOSPITAL | 1952 |
| LOCATION: ORGANIZATION | 5 |
| LOCATION: STREET | 1492 |
| LOCATION: CITY | 1566 |
| LOCATION: STATE | 1463 |
| LOCATION: COUNTRY | 4 |
| LOCATION:ZIP | 1519 |
| LOCATION: OTHER | 19 |
| AGE | 141 |
| DATE | 7665 |
| CONTACT: PHONE | 7 |
| CONTACT: FAX | 0 |
| CONTACT: EMA IL | 0 |
| CONTACT:URL | 0 |
| CONTACT: IPADDRESS | 0 |
| ID: SSN | 0 |
| ID: MEDICALRECORD | 2090 |
| ID: HEALTHPLAN | 0 |
| ID: ACCOUNT | 0 |
| ID: LICENSE | 0 |
| ID: VEHICLE | 0 |
| ID: DEVICE | 0 |
| ID: BIOID | 1 |
| ID: IDNUM | 6860 |
| OTHER | 0 |

Supplementary Table 2.PHI subcategory distribution

|  |  | **All settings (n=2100)** | **Setting1(n=700)** | **Setting2(n=700)** | **Setting3 (n=700)** | |
| --- | --- | --- | --- | --- | --- | --- |
|  |  |  |  |  | **PhysioNet DeID vs Annotator 1** | **PhysioNet DeID vs Annotator 2** |
| Overall | P | 0.9482 | 0.9565 | 0.9329 | 0.9263 | 0.9618 |
|  | R | 0.9445 | 0.9552 | 0.9346 | 0.824 | 0.8455 |
|  | F | 0.9464 | 0.9559 | 0.9337 | 0.8721 | 0.8999 |
| HIPAA | P | 0.9922 | 0.9989 | 0.9779 | 0.9742 | 0.9851 |
|  | R | 0.978 | 0.9843 | 0.9681 | 0.962 | 0.9554 |
|  | F | 0.9851 | 0.9915 | 0.9729 | 0.9681 | 0.97 |
| NAME | P | 0.9899 | 0.9971 | 0.9785 | 0.9563 | 0.9704 |
|  | R | 0.9773 | 0.9717 | 0.9803 | 0.729 | 0.7293 |
|  | F | 0.9836 | 0.9843 | 0.9794 | 0.8273 | 0.8328 |
| LOCATION | P | 0.8465 | 0.8583 | 0.8315 | 0.9271 | 0.9763 |
|  | R | 0.9207 | 0.9375 | 0.9099 | 0.8141 | 0.9233 |
|  | F | 0.882 | 0.8962 | 0.8689 | 0.8669 | 0.9491 |
| DATE | P | 0.9947 | 0.9996 | 0.984 | 0.9667 | 0.9844 |
|  | R | 0.9751 | 0.9786 | 0.9721 | 0.9518 | 0.9446 |
|  | F | 0.9848 | 0.989 | 0.978 | 0.9592 | 0.9641 |
| ID | P | 0.9792 | 0.9916 | 0.9493 | 0.8576 | 0.9145 |
|  | R | 0.9044 | 0.9358 | 0.8728 | 0.8519 | 0.8251 |
|  | F | 0.9403 | 0.9629 | 0.9094 | 0.8547 | 0.8675 |
| AGE | P | 0.9914 | 0.9677 | 1 | 0.8 | 0.9636 |
|  | R | 0.8099 | 0.75 | 0.907 | 0.9565 | 0.8983 |
|  | F | 0.8915 | 0.8451 | 0.9512 | 0.8713 | 0.9298 |
| CONTACT | P | 0 | 0 | 0 | 0 | 0 |
|  | R | 0 | 0 | 0 | 0 | 0 |
|  | F | 0 | 0 | 0 | 0 | 0 |
| PROFESSION | P | 0 | 0 | 0 | 0 | 0 |
|  | R | 0 | 0 | 0 | 0 | 0 |
|  | F | 0 | 0 | 0 | 0 | 0 |
| OTHER | P | 0 | 0 | 0 | 0 | 0 |
|  | R | 0 | 0 | 0 | 0 | 0 |
|  | F | 0 | 0 | 0 | 0 | 0 |

Supplementary Table 3. IAA per PHI category. IAA for all settings column was calculated by combining annotations from both annotators in all settings. In other words, IAA is calculated between (Setting 1 Annotated by Annotator 1 + Setting 2 Annotated by Annotator 1 + Setting 3 PhysioNet DeID annotations reviewed by Annotator 1) and (Setting 1 Reviewed by Annotator 2 + Setting 2 Annotated by Annotator 2 + Setting 3 PhysioNet DeID annotations reviewed by Annotator 2). The HIPAA category is a combination of various PHI categories and subcategories as per the HIPAA guidelines^[^[^8^](#_ENREF_8)^,^ [^10^](#_ENREF_10)^].^

|  |  | **All settings** | | **Setting1** | | **Setting2** | | **Setting3** | |
| --- | --- | --- | --- | --- | --- | --- | --- | --- | --- |
|  |  | **Annotator1** | **Annotator2** | **Annotator1** | **Annotator2** | **Annotator1** | **Annotator2** | **Annotator1** | **Annotator2** |
| Overall | P | 0.954 | 0.997 | 0.9564 | 0.9998 | 0.9508 | 0.991 | 0.9549 | 0.9934 |
|  | R | 0.9466 | 0.9931 | 0.9552 | 1 | 0.9411 | 0.979 | 0.9436 | 0.9934 |
|  | F | 0.9503 | 0.995 | 0.9558 | 0.9999 | 0.9459 | 0.985 | 0.9492 | 0.9934 |
| HIPAA | P | 0.9977 | 0.9961 | 0.9989 | 1 | 0.9949 | 0.9879 | 0.9993 | 0.9929 |
|  | R | 0.9819 | 0.9944 | 0.9843 | 1 | 0.9799 | 0.9828 | 0.9814 | 0.9929 |
|  | F | 0.9897 | 0.9952 | 0.9915 | 1 | 0.9873 | 0.9854 | 0.9903 | 0.9929 |
| NAME | P | 0.9949 | 0.9991 | 0.9971 | 1 | 0.9935 | 0.9971 | 0.9941 | 0.9894 |
|  | R | 0.9779 | 0.9946 | 0.9717 | 1 | 0.9818 | 0.9836 | 0.98 | 0.9789 |
|  | F | 0.9863 | 0.9968 | 0.9843 | 1 | 0.9876 | 0.9903 | 0.987 | 0.9841 |
| LOCATION | P | 0.8489 | 0.9994 | 0.8578 | 0.9994 | 0.8396 | 0.9904 | 0.8487 | 1 |
|  | R | 0.9225 | 0.9968 | 0.9375 | 1 | 0.9156 | 0.987 | 0.9141 | 1 |
|  | F | 0.8842 | 0.9958 | 0.8959 | 0.9997 | 0.876 | 0.9887 | 0.8802 | 1 |
| DATE | P | 0.9995 | 0.9963 | 0.9996 | 1 | 0.9988 | 0.9968 | 1 | 1 |
|  | R | 0.9763 | 0.9953 | 0.9786 | 1 | 0.9756 | 0.9856 | 0.9746 | 1 |
|  | F | 0.9877 | 0.9971 | 0.989 | 1 | 0.9871 | 0.9912 | 0.9871 | 1 |
| ID | P | 0.9916 | 0.993 | 0.9916 | 1 | 0.9865 | 0.9792 | 0.9967 | 0.9859 |
|  | R | 0.9095 | 0.9861 | 0.9358 | 1 | 0.8886 | 0.9592 | 0.9052 | 1 |
|  | F | 0.9488 | 0.9896 | 0.9629 | 1 | 0.935 | 0.9691 | 0.9487 | 0.9929 |
| AGE | P | 0.9914 | 0.993 | 0.9677 | 1 | 1 | 0.9767 | 1 | 1 |
|  | R | 0.8156 | 1 | 0.75 | 1 | 0.9286 | 1 | 0.7797 | 1 |
|  | F | 0.8949 | 0.9965 | 0.8451 | 1 | 0.963 | 0.9882 | 0.8762 | 1 |
| CONTACT | P | 0 | 0.875 | 0 | 1 | 0 | 0.8333 | 0 | 0 |
|  | R | 0 | 1 | 0 | 1 | 0 | 1 | 0 | 0 |
|  | F | 0 | 0.9333 | 0 | 1 | 0 | 0.9091 | 0 | 0 |
| PROFESSION | P | 0 | 0 | 0 | 0 | 0 | 0 | 0 | 0 |
|  | R | 0 | 0 | 0 | 0 | 0 | 0 | 0 | 0 |
|  | F | 0 | 0 | 0 | 0 | 0 | 0 | 0 | 0 |
| OTHER | P | 0 | 0 | 0 | 0 | 0 | 0 | 0 | 0 |
|  | R | 0 | 0 | 0 | 0 | 0 | 0 | 0 | 0 |
|  | F | 0 | 0 | 0 | 0 | 0 | 0 | 0 | 0 |

Supplementary Table 4. DS per PHI category. DS for all settings was calculated using the same approach taken for calculating IAA for all settings, explained in Supplementary Table 3.

| **Dimension** | **Metric** | **Granularity** | **All settings** | **Setting 1 vs Setting 2** | **Setting 1 vs Setting 3^** | **Setting 2 vs Setting 3^** |
| --- | --- | --- | --- | --- | --- | --- |
| Time | Total Time | Overall | <0.0001* | 0.0667 | <0.0001* | <0.0001* |
| Quality | IAA**^** | Overall | <0.0001* | <0.0001* | <0.0001* | <0.0001* |
|  |  | NAME | <0.0001* | 0.6660 | <0.0001* | <0.0001* |
|  |  | LOCATION | 0.0038* | 0.0032* | 0.0635 | 0.5684 |
|  |  | DATE | <0.0001* | 0.0548 | <0.0001* | <0.0001* |
|  |  | ID | <0.0001* | <0.0001* | <0.0001* | <0.0001* |
|  |  | AGE | 0.2645 | 0.5063 | 0.2503 | 0.8822 |
|  | DS + | Overall | <0.0001* | <0.0001* | 0.0107* | 0.2981 |
|  |  | NAME | <0.0001* | <0.0001* | 0.0200* | 0.4025 |
|  |  | LOCATION | 0.0189* | 0.0219* | 0.0152* | 0.8002 |
|  |  | DATE | <0.0001* | <0.0001* | 0.9999 | 0.4468 |
|  |  | ID | <0.0001* | <0.0001* | 0.9955 | 0.3204 |
|  |  | AGE | 0.6459 | 0.8909 | 0.6634 | 0.7303 |
|  |  | CONTACT | 0.2467 | 0.2259 | 0.9948 | 0.8782 |

Supplementary Table 5. Significance test results. For IAA and DS, individual F-measure calculated per document was used for significance tests. However, this was not possible with the time metric, as we did not capture time taken per document. Instead, overall time taken for each batch was used to calculate the significance tests.

* The difference between the 2 settings is significant.

^ For IAA, Setting 3’s F-measure was obtained by averaging the individual F-measures of PhysioNet DeID vs Annotator 1 and PhysioNet DeID vs Annotator 2.

+ For DS, in each setting, Annotator 1’s and Annotator 2’s F-measure per document was averaged.

**IAA, DS, Precision, Recall and F-measure formulas.**

IAA= DS= F-measure=(2∗Precision∗Recall)/(Precision + Recall)

Precision= (True positive)/(True positive + False positive)

Recall= (True positive)/(True positive + False negative)

Supplementary Table 6A. IAA, DS, Precision, Recall and F-measure formulas used to evaluate quality of OpenDeID corpus. We adopted micro-averaged strict setting of F-measure to report IAA and DS[[17](#_ENREF_17), [19](#_ENREF_19)]. Micro-averaged F-measure averages F-measure for each annotation throughout the corpus. Strict setting of F-measure requires exact matching of annotation offsets.

| **Sample Annotation** | | | | | |
| --- | --- | --- | --- | --- | --- |
| ANNOTATOR:  ****ADDITIONAL REPORT: HER-2 SISH RESULTS (24.7.13)*  *Blocks were sent to Steve Waugh Hospital for Her2 ISH analysis, Dr M Waugh from reports:*  *HER2 IHC Result: 2+*  *Processed at POW pathology.*  GOLD SET:  ****ADDITIONAL REPORT: HER-2 SISH RESULTS (24.7.13)*  *Blocks were sent to Steve Waugh Hospital for Her2 ISH analysis, Dr* ***M Waugh*** *reports:*  *HER2 IHC Result: 2+*  *Processed at* ***POW pathology****.* | | | | | |
| **Sample calculation of true positives, false positives, and false negatives** | | | | | |
| **Annotator 1 or 2** | **Gold** | **TP** | **FP** | **FN** | **Remarks** |
| *24.7.13* |  |  |  |  | This is a FP because annotator annotated it is a date due to the format observed. However, according to the gold set this entity identified is a standard test code used by the pathology and as such not PHI. |
| *Steve Waugh Hospital* | *Waugh Hospital* |  |  |  | This is a FP and FN because the text boundaries do not match |
| *M Waugh* | *M Waugh* |  |  |  | This is a TP because it exists in the annotations by annotator and gold set. |
|  | *POW* |  |  |  | This is a FN because it exists only in the gold set. |

Supplementary Table 6B. Sample modified annotations of few sentences from OpenDeID corpus. These sentences annotated by an annotator are compared with the gold set to calculate true positives, false positives, and false negatives. Please note we employed strict matching which requires exact match of offsets.
